# Dopamine transporter SPECT imaging in Parkinson’s disease and parkinsonian disorders

**DOI:** 10.3906/sag-2008-253

**Published:** 2021-04-30

**Authors:** Ümit Özgür AKDEMİR, Ayşe BORA TOKÇAER, Lütfiye Özlem ATAY1

**Affiliations:** 1 Department of Nuclear Medicine, Faculty of Medicine, Gazi University, Ankara, Turkey; 2 Department of Neurology, Faculty of Medicine, Gazi University, Ankara Turkey

**Keywords:** Dopamine transporter, 123I-ioflupane, SPECT, Parkinson’s disease, parkinsonian disorders

## Abstract

The dopamine transporter (DAT) imaging provides an objective tool for the assessment of dopaminergic function of presynaptic terminals which is valuable for the differential diagnosis of parkinsonian disorders related to a striatal dopaminergic deficiency from movement disorders not related a striatal dopaminergic deficiency. DAT imaging with single-photon emission computed tomography (SPECT) can be used to confirm or exclude a diagnosis of dopamine deficient parkinsonism in cases where the diagnosis is unclear. It can also detect the dopaminergic dysfunction in presymptomatic subjects at risk for Parkinson’s disease (PD) since the reduced radiotracer binding to DATs in striatum is already present in the prodromal stage of PD. This review covers the rationale of using DAT SPECT imaging in the diagnosis of PD and other parkinsonian disorders, specifically focusing on the practical aspects of imaging and routine clinical indications.

## 1. Introduction

Parkinson’s disease (PD) is characterized by the progressive loss of dopaminergic neurons in the pars compacta of the substantia nigra [1,2]. Although the pathophysiologic mechanisms remain unclear, PD is a neurodegenerative disease that results in decreased striatal dopamine production and causes the symptoms of parkinsonism (rigidity and bradykinesia) when 60%–80% of the presynaptic dopaminergic neurons are lost [2,3]. The dopamine transporter (DAT) proteins, which are found in the membrane of presynaptic endings of dopaminergic neurons and responsible for the reuptake of dopamine from the synaptic cleft, reflect the degree of presynaptic dopaminergic neuron loss and may be used as a diagnostic biomarker of PD [1,2,4]. The level of DAT expression in the striatum (caudate and putamen) can be evaluated by several tropane-based radiotracers, among which iodine-123 (123I)-FP-CIT (123I-ioflupane) and 123I-beta-CIT are the commercially available and the most commonly used ones [5–7]. Brain single-photon emission tomography (SPECT) is the modality to assess DAT expression in the striatum. Positron emission tomography (PET) radiotracers for DAT imaging [such as fluorine-18 (18F)-FP-CIT] are also present, however, none of them are commercially available yet [4].

The differential diagnosis of PD involves a mixed group of diseases which are collectively called as parkinsonian syndromes and may be functionally divided into two groups: In the first group are the parkinsonian disorders related to a striatal dopaminergic deficiency which include the neurodegenerative atypical parkinsonism syndromes, namely multiple system atrophy (MSA), progressive supranuclear palsy (PSP), cortical-basal ganglionic degeneration (CBD), and Lewy body dementia (LBD) [4]. The other group involves movement disorders that are not related to a striatal dopaminergic deficiency, such as essential tremor (ET), adult-onset dystonic tremor, secondary parkinsonism (due to dopamine receptor blocking drugs and pallidal toxins), vascular parkinsonism, normal pressure hydrocephalus and psychogenic parkinsonism [4]. Therefore, DAT SPECT imaging may be useful for the differential diagnosis of parkinsonism by providing evidence for or ruling out the presence of striatal dopaminergic deficiency [1,2,4–9]. In clinical practice, DAT SPECT imaging may contribute to clinical management of patients who do not fully meet the diagnostic criteria of PD or who have atypical findings, do not give an adequate response to treatment and show mild symptoms at an early stage. 

Both presynaptic and postsynaptic functions of the dopaminergic neurotransmitter system can be evaluated with nuclear medicine methods. While presynaptic dopaminergic imaging is used to investigate the presence of a neurodegenerative process in the dopaminergic system, postsynaptic dopaminergic imaging is mostly used to make a differential diagnosis of PD with the neurodegenerative atypical parkinsonism syndromes [1,2,4–10]. However, the clinical uses of postsynaptic D2-receptor imaging with SPECT or PET are not common today. In addition to the limited availability of D2-receptor radiotracers, the widely available brain 18F-fluorodeoxyglucose (FDG) PET imaging has a higher diagnostic accuracy than postsynaptic D2-receptor SPECT imaging in the differential diagnosis of PD with the neurodegenerative atypical parkinsonism syndromes [11]. Myocardial scintigraphy of 123I-metaiodobenzylguanidine (mIBG) is another method with high diagnostic accuracy in the differential diagnosis of PD with the neurodegenerative atypical parkinsonism syndromes (MSA, PSP, and CBD) [12]. 

DAT SPECT studies were carried out in the Gazi University Hospital for the first time in our country within the scope of the multicenter ENC-DAT project launched in 2009 by the European Association of Nuclear Medicine (EANM) neuroimaging committee. The project aimed to create a normative 123I-ioflupane SPECT database [13–17]. Then, the first clinical routine DAT SPECT studies in our country were carried out in our center. Today, as the clinicians’ awareness of this practice is increasing, DAT SPECT studies are becoming widespread in our country. Therefore, we aimed to share our experience and summarize the practical aspects and the clinical role of DAT SPECT imaging in the evaluation of patients with parkinsonism in this review. 

## 2. Practice of DAT imaging

### 2.1. Radiotracers 

The basic structure of a radiotracer is composed of a bioactive molecule that binds to the molecular target and a radionuclide that can be detected by the SPECT or PET camera. The most commonly used radionuclides for SPECT imaging are 123I and technetium-99m (99mTc) that both emit gamma rays of low-energy and have physical half-lives of approximately 13 and 6 h, respectively. Due to the higher target binding ratios and the presence of quantitative analysis tools that include normal reference data, the DAT radiotracers that are labeled with 123I, especially 123I-ioflupane, are generally preferred for presynaptic dopaminergic imaging [5–9]. The PET radiotracers for dopaminergic imaging are radiolabelled with 18F or carbon-11 (11C) that have 110 and 20 min of physical half-lives, respectively. Since the physical half-lives of PET radiotracers are relatively short and the production costs are higher when compared to SPECT radiotracers, their use is mostly limited to research centers with cyclotron facilities.

In vivo measurement of DAT availability is possible by using several DAT specific SPECT and PET radiotracers (Table 1) [5–9]. DAT is among the several targets in the striatal dopaminergic system that can be studied with nuclear medicine methods. The alternative radiotracers target the aromatic-L-amino acid decarboxylase (AADC) enzyme in the presynaptic dopaminergic neuron and the vesicular monoamine transporter protein 2 (VMAT2) in the membrane of presynaptic dopaminergic neuron’s storage vesicles. Dopamine is produced as a result of a two-step synthesis using L-tyrosine in the presynaptic dopaminergic neuron: In the first step, L-dopa is formed by hydroxylation of L-tyrosine and in the second step dopamine is formed by the effect of AADC on L-dopa. This dopamine is then stored in vesicles at the presynaptic neuron terminal via the VMAT2. When the presynaptic dopaminergic neuron is stimulated, the dopamine in the vesicles is discharged into the synaptic space. Dopamine in the synaptic space can be taken back into the cell by the DAT in the membrane of the presynaptic neuron. For the imaging of postsynaptic dopaminergic neurons, several radiotracers that target dopamine receptors (D1-, D2-receptors) are also available [10]. Since more than 90% of D2-receptors are found in the postsynaptic cell, radiotracers that bind to D2-receptors are used to evaluate the functions of these cells [10].

**Table 1:** The SPECT and PET radiotracers used for the evaluation of striatal dopaminergic neurotransmission in PD and parkinsonian disorders [4–8, 10].

Molecular target in the striatum	SPECT radiotracers	PET radiotracers	Common indications
Presynaptic dopaminergic terminal			
DAT	99mTc-TRODAT,123I-altropane,123I-beta-CIT,123I-FP-CIT (ioflupane)	18F-FP-CIT,11C-PE2I,18F-FE-PE2I	Evaluation of functional integrity of striatal dopaminergic neurons in patients with clinically uncertain parkinsonian syndromes
VMAT2		11C-DTBZ,18F-FP-DTBZ	
AADC		18F-fluorodopa	
Postsynaptic dopaminergic nerve			
D2 receptors	123I-IBZM	11C-raclopride,18F-fallypride,18F-DMFP	Differential diagnosis of parkinsonian syndromes (PD vs. MSA, PSP and CBD)

Among the various SPECT and PET radiotracers for the functional imaging of the dopaminergic system, only 123I-ioflupane and 123I-beta-CIT have received both the U.S. Food and Drug Administration (FDA) and the European Medicines Agency (EMA) approvals. An alternative for DAT SPECT imaging is PET imaging with 18F labeled fluorodopa. Although the 18F-fluorodopa has been approved by EMA, it has not yet been approved by the FDA for production and commercial distribution. None of the other radiotracers used in dopaminergic imaging has yet gone through the aforementioned approval processes. In Turkey, 123I-ioflupane is currently the only radiotracer that is approved by the Turkish Medicines and Medical Devices Agency of the Ministry of Health. Today, DAT SPECT study is the most commonly used molecular imaging method in parkinsonian disorders due to the presence of long-term clinical experience, normative data that have been created, and the practice guidelines which were prepared by several medical specialty associations [5–9, 17–22].

### 2.2. Patient preparation

Patients should avoid taking any drugs or other psychotropic substances which may significantly influence the DAT binding of radiotracers before the investigation (Table 2) [5,6,8]. The recommended withdrawal period is at least five times the drug’s biological half-life [5,6,8,23]. The antiparkinsonian medications (such as L-dopa, dopamine agonists, NMDA receptor blockers, MAO-B, and COMT inhibitors) do not need to be stopped since they do not significantly affect DAT binding [5,6,8,23]. A recent metaanalysis showed that the striatal DAT availability was decreased in cocaine, amphetamine, and methamphetamine users [24]. In the same metaanalysis, it was shown that smoking does not cause a significant difference in striatal DAT availability. Regarding sedatives, another metaanalysis showed that opioid users had a significantly lower DAT availability and alcohol users had no significant difference in DAT availability [25]. Other drug groups that may alter DAT binding of radiotracers include opioid derivatives (fentanyl, modafinil), central nervous system stimulants (ephedrine, phentermine), amphetamines (methylphenidate), antidepressants (bupropion, mazindol, radafaxine), adrenergic agonists (norepinephrine, phenylephrine), anticholinergic drugs (benztropine), and anesthetics (isoflurane, ketamine, phencyclidine) [5,6,8,23,24]. Besides, selective serotonin reuptake inhibitors may slightly increase the binding of radiotracers to DAT [26,27]. These effects, which should be taken into consideration in clinical trials, do not make a difference in clinical routine practices to the extent that they affect the patient’s imaging results [23]. Cholinesterase inhibitors and neuroleptics also do not affect the DAT binding of radiotracers [23].

**Table 2:** Table 2. Drugs or other psychotropic substances that can significantly affect radiotracer binding in the DAT SPECT study [5–8, 23–26].

Drug class	Drug names	Potential effects
Cocaine	Cocaine	striatal 123I-ioflupane binding
Amphetamines	Amphetamine, methamphetamine, methylphenidate	striatal 123I-ioflupane binding
CNS stimulants	Phentermine, ephedrines	striatal 123I-ioflupane binding
Opioid derivatives	Fentanyl, modafinil	striatal 123I-ioflupane binding
Antidepressants	Bupropion, mazindol, radafaxine	striatal 123I-ioflupane binding
Adrenergic agonists	Norepinephrine, phenylephrine	striatal 123I-ioflupane binding. This effect occurs especially when infused at high doses
Anticholinergic drugs	Benztropine	striatal 123I-ioflupane binding. Other anticholinergic drugs striatal 123I-ioflupane binding to a degree that does not affect visual evaluation
Anesthetics	Isoflurane, ketamine, phencyclidine	striatal 123I-ioflupane binding

It is necessary to minimize free 123I accumulating in the thyroid gland by blocking the thyroid gland with Lugol solution (equivalent to 100 mg iodide) or potassium perchlorate (600 mg) at least 1 h before radiotracer injection [5–9]. In patients who are sensitive to any of these products, the use of these products should be avoided. Even in the absence of a blocking agent, the radiation dose to which the thyroid is exposed will be low [9]. In some patients, an antiallergic premedication can be given before the procedure. However, iodine allergy in the patient is not a contraindication for radiotracer administration.

Pregnancy is a contraindication for the DAT SPECT study. Breastfeeding is a relative contraindication and patients should discontinue breastfeeding for 6 days after they receive the 123I labeled radiotracer. Fasting is not required and patients should be encouraged to be well hydrated on the day of examination to minimize bladder radiation exposure since the radiotracer is excreted in the urine. 

It is necessary to control and ensure that the patient will be able to cooperate and lie still for approximately 40 to 60 min during the investigation. If sedation is required, it should not be given earlier than 1 hour before the SPECT acquisition.

### 2.3. Safety of DAT imaging

In adults, the recommended dose is 110–250 MBq (usually 185 MBq) for 123I-labeled DAT radiotracers. The radiotracer will be delivered ready to use and should be injected within the time period specified by the manufacturer, usually on the day of delivery. Radiotracer is injected in the form of a slow i.v. bolus that lasts about 20 s.

A radiotracer dose of 185 MBq will cause an effective dose of approximately 4.4 mSv in an adult patient [9]. This radiation dose is higher in comparison to radiographic examinations (with average effective doses of 0.1–1.5 mSv) and lower in comparison to a chest computerized tomography examination (with an average effective dose of 8 mSv) [28]. Bladder wall (organ receiving the highest radiation dose) will be exposed to a radiation dose of approximately 1.0 rad [9]. 

In a safety analysis of clinical studies that involved more than a thousand patients, mild side effects such as headache, nausea, dizziness, nasopharyngitis, and development of hematoma at the injection site were observed in less than 4% of patients after 123I-ioflupane injection [29]. No serious adverse effects were observed related to 123I-ioflupane administration and the radiotracer was well tolerated. 

There is no established clinical indication for DAT SPECT for children and the safety of the use of these radiotracers in pediatric patients has not been established. The effect of kidney or liver failure on DAT SPECT imaging is unknown. Since I-123 FP-CIT is excreted by the kidneys, patients with severe renal impairment may have an increased dose of radiation and a possible change in DAT SPECT images [9,29].

### 2.4. SPECT imaging

SPECT imaging should be carried out by or under the supervision of nuclear medicine physicians. It is preferable to perform DAT examination on SPECT cameras with multiple detectors. Single-detector SPECT systems are not recommended, since acquiring sufficient counts will require long imaging times, which may make examination difficult for the patient and cause movement artifacts [5–9].

The recommended imaging time for 123I-ioflupane is 3 to 6 h after the radiotracer injection. For 123I-beta-CIT, this period is 18 to 24 h after injection. It is recommended to standardize imaging times in a center (for example, 3 h after injection for 123I-ioflupane) so that images of different patients or follow-up images of a patient will be more comparable by excluding time-dependent changes in radiotracer uptake in the brain [5–9].

The distribution of DAT radiotracers in the brain is not affected by functional activations. Therefore, patients do not need to remain in a quiet/dim environment before and during the injection of the radiotracer. Similarly, there are no dietary restrictions for the DAT SPECT study. If the patient has claustrophobia or if the patient is unable to lie still in the camera, a short acting benzodiazepine (sedative) can be given before imaging [5–9].

Technical specifications on how to perform DAT SPECT imaging are excluded from the scope of this article since they can be found in detail in the current procedure guidelines [5–9]. Some important practical issues related to DAT SPECT imaging are as follows: DAT SPECT imaging will take approximately 30–40 min on a two-detector gamma camera. The acquired projection images should be reviewed in terms of patient motion before image processing. Given the limited spatial resolution (about 8–10 mm) of SPECT images, small amounts of patient motion can be tolerated. However, if the patient is observed to move significantly, it is recommended to repeat the imaging. Iterative reconstruction methods are generally preferred for obtaining cross-sectional SPECT images from the projection data since physical corrections (such as attenuation, scatter corrections) can be added to the reconstruction algorithm. Also, when using normal databases for semiquantitative evaluation of DAT SPECT images, care should be taken to match the reconstruction parameters with the parameters which were used to create the normal database. Performing physical corrections improves the accuracy of quantitative analysis and can significantly affect the values ​​obtained from quantitative analysis [14]. In general, SPECT systems are subjected to routine quality control procedures within the framework of a quality control program. Besides, imaging of an anthropomorphic striatal brain phantom by applying the routine clinical imaging and processing parameters used in DAT SPECT studies can be useful to assess the system’s overall SPECT image quality.

## 3. Assessment of DAT SPECT images

The assessment of DAT binding in striatum depends on visual interpretation of SPECT images and the results of quantitative analysis of SPECT data, if available. The visual assessment aims to decide whether the DAT binding is normal or abnormal. If DAT binding is abnormal, the degree of decrease in DAT binding in striatal subregions regarding left-to-right asymmetry and posterior-to-anterior uptake ratio (gradient) should be evaluated [5, 6]. Given the age-dependent decrease of striatal DAT binding in normal populations, quantitative analysis of DAT SPECT is strongly recommended for an objective assessment [5, 6]. In clinical trials, quantitative analysis is often used to measure DAT intensity as it has high reproducibility and objectivity to determine the degree of disease and response to treatment [14,26,30,31]. In various studies, it has been shown that the inclusion of quantitative analysis results in the image evaluation process of readers increases the diagnostic performance [15, 30–32].

### 3.1. Visual assessment

When preparing DAT SPECT images for visual evaluation, it is recommended to determine the axial plane in a standard way which is parallel to the line passing between the anterior and posterior commissures. DAT SPECT images should be evaluated on the computer screen since the color scale and contrast can be manipulated by the reader. In the presence of an anatomical lesion and cerebral atrophy, the location or shape of striatal structures may change. Therefore, when evaluating DAT SPECT images, computed tomography (CT) and magnetic resonance imaging (MRI) findings should be taken into account. Brain MRI findings can also help assess vascular comorbidity [9].

**Figure 1:**
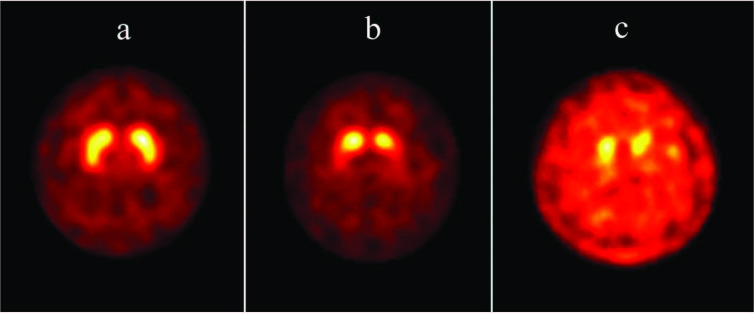
Axial 123I-ioflupane DAT SPECT images of three different patients with parkinsonism. (a) Striatal radiotracer uptake is visually symmetrical and normal. The background activity is low. Both striata have comma shape and sharp borders. The findings of the quantitative analysis were in normal ranges. These findings exclude the presence of a striatal dopaminergic deficiency. (b) Radiotracer uptake in putamen is bilaterally decreased and background activity is slightly increased. Both striatal structures took an oval appearance. Specific binding ratios calculated from both putamens were significantly lower than the patient’s age group. The asymmetry index was 25%. Specific binding ratios calculated from the caudate nuclei were also lower than the mean values determined for the patient’s age group, but this difference was not statistically significant (within the 95% confidence interval). These findings indicate a moderate nigrostriatal dopaminergic neuron loss and the pattern of involvement may be consistent with early-stage PD. (c) Striatal radiotracer uptake is decreased and background activity is increased significantly. Specific binding ratios calculated from both putamens and caudate nuclei are significantly lower than the patient’s age group. These findings indicate a severe degree of nigrostriatal dopaminergic neuron loss and the pattern of involvement may be consistent with late-stage PD.

It has been shown in various studies that experienced readers can read DAT SPECT images with high accuracy only by visual evaluation [20,30,33,34]. Visual evaluation is usually sufficient to evaluate striatal left-to-right asymmetry and differential radiotracer uptake in striatal subregions. In axial sections, normal striatum should be in the form of a comma and its borders should be clearly visible (Figure 1a). A slight asymmetry in striatal radiotracer uptake may be seen in normal individuals. The findings of quantitative analysis may be useful when evaluating the degree of striatal asymmetry. In addition, the level of striatal radiotracer uptake should be compared with background activity. A good contrast is expected between the striatal and the background signals. However, there is some decrease in striatal radiotracer uptake in both caudate and putamen with normal aging. In this regard, quantitative analysis can help the reader in evaluating the findings.

Decreasing striatal radiotracer uptake and increasing background activity in DAT SPECT images is an indicator of decreased presynaptic dopaminergic function. Striatal involvement can occur unilaterally or bilaterally. In PD, the most prominent decrease of radiotracer uptake occurs in the dorsal putamen contralateral to the side where neurological findings are more evident. With the progression of the disease, the involvement of anterior putamen and then the caudate nucleus becomes more evident. Although this posterior-to-anterior gradient is also seen in the later stages of the disease, it is more evident in the early stages [1,33]. Since putamen is often more severely affected in early-stage PD (such as in patients with premotor or hemiparkinsonism findings), the striatum typically has an oval or circular appearance on DAT SPECT images (Figure 1b) [18,33,35,36]. Background activity increases more as a result of disease progression and decreasing striatal radiotracer uptake (Figure 1c). DAT SPECT imaging has a high sensitivity in the early stages of PD and the high negative predictive value is an important advantage of this method [30,33]. Indeed, in a controversial group of patients named SWEDD (scan without evidence of dopaminergic deficit), who were clinically diagnosed with PD but had normal DAT SPECT scans, the findings did not change during the clinical follow-up and patients did not show progression [37,38]. This observation indicates that the specificity of DAT SPECT imaging for early-stage PD may be higher than the clinical evaluation.

In patients with PD, the levels of DAT binding measured with PET or SPECT will overestimate the true degree of neurodegeneration in the striatum because of a downregulation of DAT expression in the remaining neurons as an adaptive mechanism to preserve synaptic dopamine levels [39,40]. On the contrary, the AADC expression as shown by 18F-fluorodopa PET imaging will underestimate the true degree of neurodegeneration because of its upregulation [39,40]. Therefore, in early PD, the striatal dysfunction indicated by DAT SPECT is more pronounced than the dysfunction observed with 18F-fluorodopa PET [40]. However, in studies comparing these two methods in the same patients, both methods had high accuracy in differentiating PD patients from healthy controls [41,42]. There is a correlation between the degree of motor impairment (Unified Parkinson’s Disease Rating Scale motor scores, Hoehn and Yahr stage scores) in early stage PD and the degree of striatal involvement in DAT SPECT imaging [40,43]. This correlation disappears in the later stages of the disease as the DAT SPECT findings reach saturation [36].

**Figure 2:**
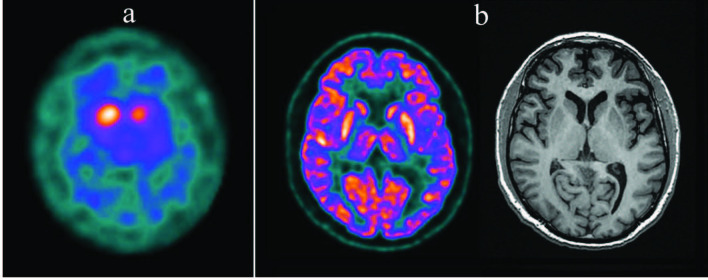
Axial 123I-ioflupane DAT SPECT and brain 18F-FDG PET images of a patient with suspected neurodegenerative parkinsonism. (a) In the 123I-ioflupane DAT SPECT examination radiotracer uptake in basal ganglia is bilaterally decreased and background activity is slightly increased. Both striatal structures took an oval appearance. Specific binding ratios calculated from both putamens and caudate nuclei were significantly lower than the patient’s age group. The decrease in uptake was more prominent in left basal ganglion and asymmetry indices were 10% and 37% for putamen and caudate nucleus, respectively. (b) The axial brain 18F-FDG PET and T1-weighted MR images which were simultaneously acquired on a hybrid PET-MR camera showed bilateral relatively increased putaminal glucose metabolism and normal findings in the cerebral cortex which is defined as a metabolic marker for PD on brain 18F-FDG PET [46]. MR examination did not show a specific pathological change. Taken together, these findings supported the diagnosis of PD in this patient.

**Figure 3:**
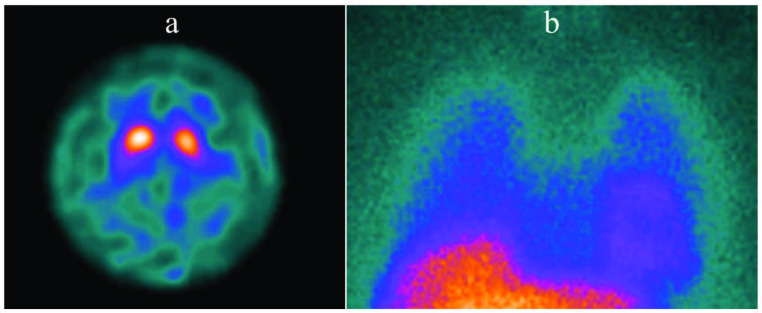
Axial 123I-ioflupane DAT SPECT and planar cardiac 123I-MIBG images of a patient with suspected neurodegenerative parkinsonism. (a) In the 123I-ioflupane DAT SPECT examination radiotracer uptake in basal ganglia is bilaterally decreased and background activity is slightly increased. Both striatal structures took an oval appearance. Specific binding ratios calculated from both putamens and caudate nuclei were significantly lower than the patient’s age group. The decrease in uptake was more prominent in left basal ganglion and asymmetry indices were 13% and 10% for putamen and caudate nucleus, respectively. The patient had clinical findings of parkinsonism and autonomic dysfunction and the presumed diagnosis was PD or MSA. Although DAT SPECT findings verified the presence of a striatal dopaminergic neurodegeneration, it was not adequate for making a differential diagnosis between PD and MSA. Therefore, a cardiac 123I-MIBG examination was requested. (b) The planar cardiac 123I-MIBG image displays normal radiotracer uptake in the left ventricle. Since cardiac 123I-MIBG uptake is expected to be decreased in PD and normal in MSA, these findings supported the diagnosis of MSA in this patient [12].

In patients with LBD, the caudate nuclei show early involvement which may cause the gradient between the putamen and caudate to be less pronounced than in patients with PD [20,30]. Similarly, the involvement of the anterior striatum is seen earlier and more prominently in parkinsonian type MSA and PSP compared to PD [40,44]. DAT function in cerebellar type MSA is higher when compared to parkinsonian type MSA [40]. Patients with CBD show a marked asymmetric striatal involvement (both in the caudate nucleus and in the putamen) contralateral to the body side where clinical findings are more evident [45]. However, it should be kept in mind that there is a significant overlap between PD and neurodegenerative atypical parkinsonism syndromes in terms of DAT SPECT findings, and this examination alone may not be sufficient for differential diagnosis [20,30]. In order to make this distinction, postsynaptic D2-receptor imaging, 18F-FDG PET (Figures 2a and 2b), or cardiac 123I-mIBG SPECT studies can be performed (Figures 3a and 3b) [11,12,46]. In clinical situations such as ET, drug-induced parkinsonism, vascular parkinsonism, and psychogenic parkinsonism DAT SPECT findings are normal; and therefore, differential diagnosis of these clinical conditions from neurodegenerative parkinsonian syndromes can be made by DAT SPECT examination [4,33,47].

DAT SPECT findings are more difficult to evaluate for the diagnosis of vascular parkinsonism. Vascular lesions in the basal ganglia are common, especially in the elderly. However, these vascular lesions cause parkinsonism in a small number of patients. If the infarct does not directly involve striatal structures, the striatal DAT radiotracer uptake is usually normal or slightly reduced. A striatal infarct causes a sharply demarcated photopenic appearance in the DAT SPECT images which differs from the findings that are usually seen in neurodegenerative parkinsonian syndromes in terms of quality and morphological appearance. Evaluation of the DAT SPECT findings together with a recent brain MRI examination of the patient may assist the reader in making this distinction [48,49].

The differential diagnosis of LBD from Alzheimer’s disease (AD) can be done with high accuracy using DAT SPECT imaging [20]. While striatal binding of DAT radiotracer is usually normal or slightly low in AD, it is significantly decreased in LBD [20, 50]. DAT SPECT is included as a biomarker in the diagnostic criteria of LBD due to its approximately 20% higher specificity in comparison to clinical diagnosis [20]. However, approximately 10% of patients who meet the pathological criteria of LBD may experience normal DAT SPECT findings. DAT SPECT becomes abnormal in these patients usually within 1.5 years [51]. This finding has been associated with the heterogeneous topographic accumulation of α-synuclein in the brain: While a low-grade of α-synuclein pathology in substantia nigra was observed in these LBD patients, they had a higher grade of α-synuclein pathology in neocortical regions [50].

### 3.2. Quantitative assessment

The most commonly used quantitative analysis method in both research and clinical routine is the calculation of the ratio of striatal radiotracer uptake to background activity (Specific binding ratio = (striatal counts–background counts)/background counts) [5–9, 31]. For this purpose, regions of interest around the striatum and its subregions (caudate nucleus, putamen) need to be created. The background activity is generally measured from the cerebellum or the occipital cortex. Although the specific binding ratio is related to DAT density, it is not a parameter that directly reflects DAT density or the number of presynaptic neurons. Many biological and technical variables (age and sex of the patient, drugs used by the patient, patient’s movement during imaging, physical characteristics of the camera, variables related to SPECT imaging and processing of images, quantification algorithm, and the region of interest used) may affect this ratio [14,15,24,25]. Regions of interest can be created according to the anatomical boundaries of striatal structures, and smaller areas of interest can be used to measure from caudate nuclei, anterior and posterior putamen [52,53]. They can be drawn by hand or to reduce operator-dependent variability quantitative analysis tools that automatically draw the regions of interest can be used [52,54–57]. Additionally, brain CT and MRI images can be used to determine the boundaries of the basal ganglia and to create regions of interest [58]. The use of radiological images for this purpose may be especially useful in cases where automatic analysis methods are not used and the striatal radiotracer uptake is very low. Moreover, in order to interpret the patient’s quantitative data correctly, it is necessary to compare them with a normal database that preferably contains age appropriate reference values [17,21]. 

In addition to the specific binding ratios for caudate and putamen, different quantitative variables such as left-to-right asymmetry values ​​[Asymmetry index = (Left–Right) / (Left+Right)] and putamen-to-caudate ratios can also be calculated from DAT SPECT images. These quantitative parameters may be useful in patients where it is difficult to evaluate images and where a clear evaluation cannot be made. In particular, the putamen/caudate ratio is an important data since it is independent of the imaging variables, reconstruction algorithm, and background activity. Similarly, assessment of asymmetry between the right and left nuclei is useful for detecting early-stage disease, but mild (less than 6%) asymmetry may also occur in the striatum or striatal subregions of healthy individuals [52].

All variables obtained as a result of the quantitative analysis of DAT SPECT images depend on the imaging method used, the reconstruction algorithm, and the corrections applied. Therefore, there are no absolute normal values ​​that can be used in all conditions. Ideally, each center should image a healthy control group and generate their own normal reference values. However, since striatal DAT density varies depending on age and sex, it is generally preferred to use existing normal DAT SPECT databases and to match the method used for DAT SPECT imaging to the method used in the creation of these databases. To evaluate the performance of this matching process systematically, it is suggested to scan an anthropomorphic striatal phantom and to determine the quantitative analysis results of the system for different levels of specific striatal radiotracer uptake values [13,16].

There are two large normal databases that can be used in quantitative analysis of DAT SPECT images. The first one is the output of the ENC-DAT study that was launched by the EANM Neuroimaging committee in 2009 and included a single center from Turkey (Gazi University Hospital) among 13 different European centers [13–17]. In this study, 123I-ioflupane DAT SPECT data were recorded from 139 healthy control subjects (74 males, 65 females; age range 20–83 years, mean age 53 years). The second normal 123I-ioflupane DAT SPECT database was obtained in the PPMI (Parkinson’s progression marker initiative) project [21]. These two studies have shown that the striatal uptake of this radiotracer decreases approximately 5.5%–6.0% (0.6%/year) in each 10-year period with normal aging and the specific binding ratios differ depending on sex. In the PPMI study, it was observed that radiotracer uptake in all striatal regions decreased significantly in the 4-year follow-up of patients diagnosed with PD [21]. This decrease occurs approximately 20 times faster than the decrease observed in normal aging. These results suggest that DAT SPECT imaging may be a suitable biomarker for demonstrating PD progression.

## 4. Conclusion

In patients with suspected PD or other parkinsonian disorders, DAT SPECT imaging be used to evaluate the functional integrity of presynaptic striatal dopaminergic neurons. DAT SPECT is a valuable tool for the early diagnosis of PD and for the differential diagnosis of PD from other nondegenerative causes of parkinsonism. Normal DAT SPECT findings exclude presynaptic striatal dopaminergic insufficiency. Thus, the clinical findings of parkinsonism in these patients may be related to clinical conditions such as ET, drug-induced parkinsonism, psychogenic parkinsonism or vascular parkinsonism. Abnormal DAT SPECT findings indicate a variety of diseases that have presynaptic striatal dopaminergic insufficiency as a common pathophysiological process, including PD, MSA, PSP, CBD, and LBD. If the examination is used for the differential diagnosis of AD with LBD, normal DAT SPECT findings support the diagnosis of AD. In some patients, a more explicit diagnosis can be made when other molecular imaging examinations (postsynaptic D2-receptor SPECT or PET, 18F-FDG PET, cardiac 123I-mIBG scintigraphy, etc.) findings are included in the interpretation.
